# Metabolic interactions between hyperhomocysteinemia and endothelin-1 among Tunisian patients with acute coronary diseases

**DOI:** 10.1186/s40659-015-0018-7

**Published:** 2015-06-24

**Authors:** Abdelkader Chalghoum, Yosri Noichri, Ines Karkouch, Azza Dandana, Bruno Baudin, Guieder Jeridi, Salima Ferchichi, Abdelhédi Miled

**Affiliations:** Laboratory of Biochemistry, Farhat HACHED Hospital, Street Doctor Moreau, 4000 Sousse, Tunisia; Valorization and Research Support Space, Biotechnology Center of Borj Cedria, 2052 Hamam Lif, Tunisia; Department of Biochemistry, Saint-Antoine Hospital, 184 Faubourg Saint-Antoine, 75571 Paris, Cedex 12 France; Department of Cardiology, Farhat HACHED Hospital, Street Doctor Moreau, 4000 Sousse, Tunisia

**Keywords:** Acute coronary syndrome, Endothelin-1, Hyperhomocysteinemia, Risk factors

## Abstract

**Background:**

Acute coronary syndromes (ACS) are complex and polygenic diseases which are a real problem of public health. These syndromes require multidisciplinary studies to understand the pathogenesis mechanisms and metabolic interactions between different risk factors.This study aimed to explore the variation of two coronary risk parameters not mentioned by Framingham cohorts, hyperhomocysteinemia and endothelin-1 (ET-1) in Tunisian coronary and the study of the variation of these parameters based on various cardiac risk factors and metabolic relationship between them.To 157 coronary and 142 healthy subjects, the concentration of homocysteine was quantified by fluorescence polarization immunoassay; the concentration of ET-1 was measured by an analytical technique, the High Performance Liquid Chromatography (HPLC) coupled with mass spectrometry.

**Results:**

Our study showed that homocysteine and ET-1 were significantly higher in patients compared to healthy subjects (24.40 ± 12.5 μmol/L vs 7.44 ± 2.5 μmol/L *p* <0.00001) for homocysteine and (15.2 ± 5.3 nmol/L vs 7.1 ± 2.7 nmol/L, *p* <0.00001) for ET-1. On the other hand, homocysteine varies according to tobacco and diabetes while ET-1 depends on the sex, hypertension, smoking, obesity and dyslipidemia and a statistically negative correlation was shown between homocysteine and ET-1 in coronary patients (r = −0.66 *p* <0.00001).

**Conclusion:**

The study of the variation of these two parameters in coronary patients and metabolic exploration of the relationship between homocysteine and ET-1 according to various risk factors and the interactions between themselves facilitates the decision of therapeutic treatment.

## Background

Acute coronary syndrome (ACS) is a major cause of morbidity and mortality worldwide. Recently, an increase in the incidence of ACS has been recorded in Tunisian cardiovascular disease register. Among diabetes, hypertension, obesity and smoking, a family lipoprotein disorder such as a high level of serum apolipoprotein B (Apo B) or/and a lower level of serum apolipoprotein A-1 accounted for almost all the population attributable risk of ACS, according to the Framingham study [1, 2].

To today, hyperhomocysteinemia and endothelin-1 (ET-1) are considered two “unconventional” coronary risk markers not classified by the Framingham cohorts,

The increase in serum homocysteine (demethylated methionine) 10 μmol/L are associated with amplification of coronary risk for 80 %, independently of the origin of this increase (genetic, nutritional, drug …). This risk is related to the harmful effects of homocysteine specially on endothelium tissue [2, 3].

The same for ET-1, potent vasoconstrictor secreted by endothelial cells (21 amino acids) and heavily involved in the genesis and complications of ACS by their pro-oxidant effect, pro-aggregating effect and pro-thrombotic effect associated with the major vasoconstrictor effect [4, 5].

In this context, comes the aim of our study which is to quantify the serum concentration of homocysteine and to measure the ET-1 serum concentration in Tunisians coronary compared to healthy subjects, investigate the impact of various risk factors on serum variation of these two parameters as well as the study of the correlation between these two markers.

## Result

Clinical characteristics and biochemical parameters of patients with coronary artery syndrome and healthy group are illustrated in Table 1. No significant difference was found between the two groups for the mean of age and sex. For cardiovascular risk factors, diabetes, hypertension, smoking and dyslipidemia are greatly mentioned among patients compared with healthy subjects. Apolipoproteins disorder was observed in patients. There was a significant increase in serum Apo B level (*p* <0.00001) and a decreased serum Apo A-1 level (*p* <0.00001) compared with serum apolipoprotein levels in healthy subjects.

**Table 1:** Clinical and biochemical features of patients with ACS and controls subjects

	Patients (*n* = 157)	Control subjects (*n* = 142)	*P*
Age (x ± σ years)	64.8 ± 11.7	56.8 ± 9.4	NS
Sex			
Men (%)	121 (77 %)	111 (78.2 %)	NS
Women (%)	36 (23 %)	31 (21.8 %)	NS
BMI (kg /m2)	27.6 ± 4	23.3 ± 2.2	NS
Hypertension (%)	88	0	-
Obesity (%)	52	8.5 %	-
Diabetes (%)	64	0	-
Smoking (%)	62.4	7	-
Family cardiac history (%)	68	6	-
Personnel cardiac history (%)	66	0	-
Post menopausal women (%)	100	82	-
Dyslipidemia (%)	40	0	-
Sedentary (%)	43	11	-
Alcohol (%)	33	14	-
Glucose (x ± σ mmolL/)	9.8 ± 4.2	5.40 ± 0.84	<0.0001
TC (x ± σ mmol/L)	5.70 ± 3.1	4.60 ± 2.6	<0.001
HDL-C (x ± σ mmol/L)	1.14 ± 0.22	1.30 ± 0.41	NS
LDL-C (x ± σ mmol/L)	3.60 ± 2.16	2.80 ± 1.4	<0.001
TG (x ± σ mmol/L)	1.60 ± 0.9	1.24 ± 0.3	NS
ApoA1 (x ± σ. g/L)	1.41 ± 0.62	1.80 ± 0.2	<0.00001
ApoB (x ± σ. g/L)	1.40 ± 0.81	0.70 ± 0.2	<0.00001
Treatment			
ACA-1 inhibitors (%)	97	0	-
Statins (%)	38	0	-
Beta-Blokers (%)	33	0	-
Ca-Blokers (%)	27	0	-
Diuretics (%)	17	0	-

*ApoA* apolipoprotein A, *ApoB* apolipoprotein *B, BMI* body mass index*, HDL-C* high density lipoprotein cholesterol, *LDL-C* low density lipoprotein cholesterol, *TC* total cholesterol, *TG* triglycerides, *x* mean*, σ* standard deviation*, NS* not significant

Furthermore, homocysteinemia and ET-1 concentration were significantly elevated in patients compared to controls (*p* <0.00001, for both comparisons). Table 2 showed this variation and as well as the study of homocysteinemia and ET-1 concentration in patients according gender and cardiovascular risk factors. This investigation shows that ET-1 depends on gender, arterial hypertension, tobacco, obesity and dyslipidemia, unlike diabetes, alcohol and physical inactivity, which are not involved in this variation. For homocysteine, it only depends on tobacco and diabetes.

**Table 2:** The distribution of Homocysteinemia and, ET-1 concentration patients and healthy subjects and according to risk factors among patients

Populations and risk factors	Hcy (μmol/L)		*P*	ET-1 (nmol/L)	*P*
Population	Patients (*n* = 157)	24.40 ± 12.5	<0.00001	15.2 ± 5.3	<0.00001
	Controls (*n* = 142)	7.4 ± 2.5		7.1 ± 2.7	
Gender	Men (*n* = 121)	24.8 ± 18.2	NS	17.4 ± 4.6	<0.00001
	Women (*n* = 36)	23.1 ± 6.3		7.8 ± 2.3	
Hypertension	Yes (*n* = 138)	24 ± 8.3	NS	16.4 ± 3.3	<0.00001
	No (*n* = 19)	27.3 ± 16.7		6.6 ± 1.9	
Diabetes	Yes (*n* = 101)	29.4 ± 7.6	<0.00001	15 ± 5.1	NS
	No (*n* = 56)	15.4 ± 6.6		15.6 ± 3.8	
Tobacco	Yes (*n* = 98)	32.4 ± 11.6	<0.00001	11.6 ± 3	<0.00001
	No (*n* = 59)	11.1 ± 2		21.2 ± 6.1	
Obesity	Yes (*n* = 82)	28.2 ± 13	NS	12.2 ± 5.3	<0.001
	No (*n* = 75)	20.2 ± 5.3		18.2 ± 4	
Dyslipidemia	Yes (*n* = 63)	26.2 ± 8.2	NS	11.8 ± 1.6	<0.00001
	No (*n* = 94)	23.2 ± 7.4		17 .5 ± 4.6	
Alcohol	Yes (*n* = 52)	24.9 ± 10.6	NS	16 ± 4.1	NS
	No (*n* = 105)	24.2 ± 7.3		14.8 ± 3.8	
Sedentarity	Yes (*n* = 67)	25.7 ± 11.1	NS	15.7 ± 3.7	NS
	No (*n* = 90)	23.5 ± 7.5		14.9 ± 2.4	

*σ* standard deviation*, NS* not significant, *x* mean*, Hcy* homocysteine*, ET-1* endothelin*-1*

**Fig. 1:**
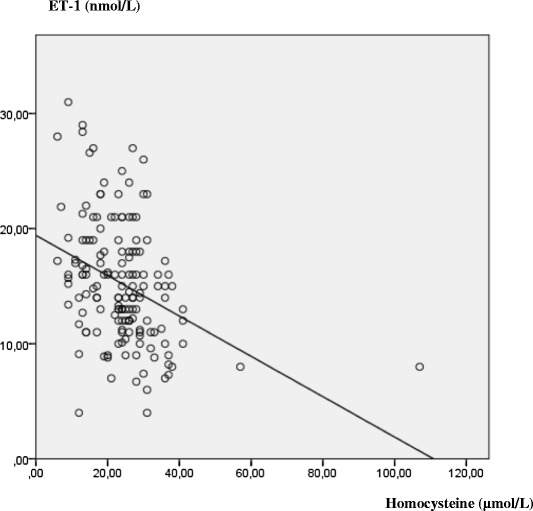
The negative correlation between homocysteine and ET-1 concentration in patients population (r = −0.66, *p* < 0.00001)

Besides to that, a negative statistically correlation was found (r = −0.66 *p* <0.00001) between homocysteinemia and ET-1 concentration (Fig. 1).

## Discussion

Varied risk factors among patients group confirm the multifactorial and polygenic origin of the ACS, well explained by the Framingham study. The lipid profile of patients was partially balanced because the hypolipidemic treatment, essentially the angiotensin-1 converting enzyme inhibitors with its beneficial effects against metabolic syndrome and lipid complications [2].

The increased of apolipoprotein B concentrations in patients consolidate its atherogenic effects and its involvement in the ACS genesis, although the majority of patients are under statins and anti-hypertensive medication. Unlike reversible dyslipidemia (only related to diet and lifestyle), easy to corrected by statins, mixed and genetic dyslipidemia requires a combined medication (statins + fibrates, or nicotinic acid) [6, 7].

Whereas, Apo A1 (statistically higher in healthy group) has a cardio-protective effect [2, 8, 9].

Statistically significant elevation of homocysteine suggests an impact of this parameter in the ACS genesis and complications. In indeed, the implication of hyperhomocysteinemia in cell injury of the vessel wall, vascular connective tissue, oxidative imbalance and prothrombotic effect is well described in various prospective studies. Hyperhomocysteinemia is strongly incriminated in the liberation of von Willebrand factor, in activation of V and VIII factors of coagulation, in inhibition of C and S proteins, in lipid peroxidation… These effects combine, and accelerate of the atherosclerotic plaque formation and contributing to subsequent thrombotic and ischemic complications [2, 9].

In literature, hyperhomocysteinemia is classified according to its severity into three types (moderate, intermediate and severe), the first with a lower concentration (<30 μmol/L), the second with a range between 30 and 100 μmol/L, and the third defined by a higher concentration (>100 μmol/L). The first type is the most dominant in the world because it is metabolic and dietary origin usually reversible and easy to corrected, while intermediates and severe hyperhomocysteinemia are the most serious and difficult to correct because they are often genetically determined (caused by homozygous mutations in the genes responsible for the degradation of homocysteine). Statistically, they are the least frequent, which is well defined in our study, with dominance of moderate hyperhomocysteinemia, a minority are intermediate or severe and they are also behind of the big variability to this marker in patients [10, 11].

Statistically significant elevation of homocysteine in smoking and obese coronary is associated with the implication of obesity and smoking in the metabolic syndrome genesis and complications, the latter is proved responsible for lipid disorder and hepatic depletion, in an advanced stage, the liver will be unable to metabolize excess homocysteine (by secretion of degradation and/or correction enzymes) [2, 12–14].

In addition, Our study showed a statistically significant increase in the ET-1 values among patients compared to control subjects, which reflects its role not only in prolonged vasoconstriction but also its atherogenic impact, its pro-oxidant effect and its pro-aggregating role [4, 15].

The high concentration of ET-1 among men patients compared to women patients shows the effect of the male gender as an un-modifiable cardiovascular risk factor.

This increase of ET-1 level is of metabolic origin and non-genetic, since our peptide is expressed by an autosomal gene (located on chromosome 6) and the same thing for cleavage and maturation enzymes (endothelin-1 converting enzyme…) are also derived from autosomal genes [16].

The elevated concentration of ET-1 among hypertensive patients compared with normotensive patients is explained by the vasoconstrictor effect of ET-1 and its roles in the sodium reabsorption. In hyperactivity, these regulatory effects become hypertensive [4].

The ET-1 concentration was inhibited by smoking, obesity and dyslipidemia, this inhibition is explained in the literature by the roles of these factors in the genesis and complications of metabolic syndrome [17]. The latter is responsible for the liver impotence in degradation and neutralization of cytotoxic elements (that infect the endothelium, main secretory tissue of ET-1). In addition, nicotine has a harmful effect on the endothelium and prevents secretion of endothelial factors [17–19].

Unlike other studies, in our population of patients, diabetes was not implicated in the change of ET-1 concentration because the drug used and the recommended diet [20, 21].

Statistically negative correlation between homocysteine and ET-1 (Fig. 1) is explained by the impact of metabolic syndrome in the liver impotence in the neutralizing of homocysteine. This demethylated methionine causes serious and irreversible damage (necrosis, apoptosis…) in the endothelium; principal secretory tissue of ET-1. With a similar mechanism, tobacco (a major actor of the metabolic syndrome) inhibits the metabolism of the homocysteine and blocks the secretion of the ET-1 due to the adverse effects of nicotine on the endothelial tissue [2, 22].

Our result is supported by the study of Drunat S et al. [23], who experimentally showed that homocysteine is responsible for the braking of the transcription of the gene which expresses the pre-pro-endothelin-1 (inactive precursor of ET-1). According to his study, an endothelial cell line treated by homocysteine has a low level of mRNA of the pre-pro-endothelin-1 compared to an untreated line.

## Conclusion

Although they are negatively correlated, hyperhomocysteinemia and increase of ET-1 concentration appears involved not only in the susceptibility to ACS, but also in the subsequent complications and in other pathologies, including kidney diseases [24]. These two parameters vary differently depending on the risk factors that reflect the complexity of ACS, even the risk markers can be negatively correlation a specific stage of the disease.

## Methods

### Populations study

This is a prospective study, in which, sampling was carried between January 2010 and November 2011. 157 Tunisian coronary subjects (121 men and 36 women) middle-aged (64.8 ± 11.7 years) were recruited from the Cardiology Service of Farhat Hached Hospital of Sousse, Tunisia. 142 healthy subjects (111 men and 31 women) middle-aged (56.8 ± 9.4 years) were considered as the healthy group.

The patients and healthy subjects signed a free and clear consent which explains the objectives of this work with an undertaking not to publish the names of participants, their personal data including test results (following the instructions of National Committee of Medical Ethics, in Tunisia, consistent with the Declaration of Helsinki).

A datasheet had been prepared for each subject (patient or healthy individual) to identify cardiovascular risk factors and to know the susceptibility degrees to ACS. This sheet contains the anthropometric characteristics, the biological parameters, the risk factors, the treatments of patients.

### Laboratory analysis

Venous blood samples were drawn after 12 h overnight fast. A collection of two tubes were made for each patient and witness: one tube without anticoagulant for Homocysteinemia, ET-1 concentration, lipid profile and apolipoprotein and the second tube with Heparin Lithium for glycemia.

A simple biochemical investigation (blood glucose, lipid profile) was done by colorimetric essay (Randox-Antrim, UK), LDL-C was determined by Friedewald formula for TG levels which does not exceed 4.5 mmol/L and apolipoproteins (Apo A1, Apo B) measurements by a immunonephelemetry essay (Cobas Integra 400, Roche) to explore the risk of exposure to acute Coronary syndrome in patients and healthy group.

The homocysteine was quantified by an immunoassay fluorescence polarization immunoassay (FPIA) (Abbott AxSYM-Diagnostics, Wiesbaden, Germany).

After plasmatic ET-1 extraction with ethanol, its concentration was measured by High Performance Chromatography, coupled to Mass Spectrometry (3200 Q TRAP LC/MS/MS system), according to the Walczak M protocol, 2010 [5], using a synthetic standard (ET-1 Sigma-Aldrich St. Louis, MO, USA) for the specific spectra identification and for the calibration curve determining. Chromatographic separation was carried out with C18 analytical column (30 mm × 2.1 mm, 3.5 μm, Waters Ireland) set at 20 °C. Two solvents mixtures were used: solvent A: Acetonitrile and solvent B: H_2_O. The following gradient was used: 0–5 min 0–100 % A; 5–7 min 100 % A; 7–8, 100-0 % A; 8–15 min, 100 % B. The flow rate was set at 300 μLmin^−1^ and a sample volume of 25 μL was injected in the analytical column.

Our biochemical analyzes were carried immediately after blood sampling, excepting the quantification of ET-1, which started in October 2012 and the samples were stored in −80 °C according to our reference protocol [5].

### Statistical analysis

Database management and statistical analyses were carried out using SPSS, version 21.0. Results are presented as means ± SD, or percentages. Means were compared using Student test. The relations between variables were assessed with Pearson’s correlation analysis. The significance threshold was set at 5 % (*p* < 0.05).

## Abbreviations

ACS: Acute coronary syndrome
Apo A1: Apolipoprotein A1
Apo B: Apolipoprotein B
ET-1: Endothelin-1
SPSS: Statistical package for the sociological sciences

## References

[1] Noichri Y, Chalghoum A, Chkioua A, Bruno B, Ernez S, Ferchichi S (2013). Low erythrocyte catalase enzyme activity is correlated with high serum total homocysteine levels in Tunisian patients with acute myocardial infarction. Diagn Pathol.

[2] Chalghoum A, Noichri Y, Chkioua L, Gammoudi I, Dandana A, Khelil S (2012). Metabolic interactions between the hyperhomocysteinemia and angiotensin-1 converting enzyme activity in Tunisian patients with coronary heart disease. Ann Biol Clin.

[3] Freitas AI, Mendonca I, Guerra G, Brión M, Reis RP, Carracedo A (2008). Methylene tetrahydrofolate reductase gene homocysteine and coronary artery disease: the A 1298 polymorphism does matter influence from a case study. Thromb Res.

[4] Sayed S, Nussberger J, Aubert F, Gohlke P (2003). Measurement of plasma endothelin 1 in experimental hypertension and in healthy subjects. Am J Hypertens.

[5] Walczak M, Fedorowicz A, Chłopicki S, Oleksiak J (2010). Determination of endothelin-1 in rats using a high-performance liquid chromatography coupled to electrospray tandem mass spectrometry. Talanta.

[6] Vega GL, Grundy SM (1998). Effect of statins on metabolism of apo-B-containing lipoproteins in hypertriglyceridemic men. Am J Cardiol.

[7] Gao F, Ballantyne C, Ma L, Virani SS, Keinan A, Brautbar A (2014). Rare LPL gene variants attenuate triglyceride reduction and HDL cholesterol increase in response to fenofibric acid therapy in individuals with mixed dyslipidemia. Atherosclerosis.

[8] Schen AJ (2004). Renin-angiotensin system inhibition prevents type 2 diabetes mellitus. Diabetes Metab.

[9] Dhahmija RK, Gaba P, Arora S, Kaintoura A, Kumar M, Bhattacharjee J (2009). Homocysteine and lipoprotein (a) correlation in ischemic stroke patients. J Neurol Sci.

[10] Kerkeni M, Added F, Ben Farhat M, Miled A, Trivin F, Maaroufi K (2008). Hyperhomocysteinaemia and parameters of antioxidative defence in Tunisian patients with coronary heart disease. Ann Clin Biochem.

[11] Chalghoum A, Noichri Y, Jaidane Z, Gammoudi I, Chahed H, Dandana A (2010). Activity of angiotensin I converting enzyme and hyperhomocysteinemia in Tunisian patients with coronary disease. Immuno Biol Spec.

[12] Mancini F, Cianciosi A, Reggiani GM, Fachinetti F, Battaghia C, Aloysio D (2009). Endothelial function its relationship to leptin homocysteine and insulin resistance in lea and overweight eumenennorrheic women and PCOS patients: a patients a pilot study. American Soc Rep Med.

[13] Kerkeni M, Letaief A, Achour A, Miled A, Trivin F, Maaroufi (2009). Hyperhomocysteimia paraoxanase concentration and cardiovascular complications in Tunisian patients with non diabetic renal disease. Clin Biochem.

[14] Koubaa N, Nakbi A, Hammami S, Attia N, Mehri S, Ben Hamda K (2009). Association of homocysteine thiolactonase activity and PON1 polymorphisms with the severity of acute coronary syndrome. Clin Biochem.

[15] Mayyas F, Al-Jarrah M, Ibrahim K, Mfady D, Van Wagoner D (2015). The significance of circulating endothelin-1 as a predictor of coronary artery disease status and clinical outcomes following coronary artery catheterization. Cardiovasc Pathol.

[16] Wang LS, Tang PN, Zhu HJ, Zhou B, Yang L, Wang B (2007). Endothelin-converting enzyme-1b C-338A polymorphism is associated with the increased risk of coronary artery disease in Chinese population. Clin Chim Acta.

[17] Hao L, Wang X, Cheng J, You J, Ma S, Zhong X (2014). The up-regulation of endothelin-1 and down-regulation of miRNA-125a-5p, −155, and -199a/b-3p in human atherosclerotic coronary artery. Cardiovasc Pathol.

[18] Matsuda M, Shimomura I (2013). Increased oxidative stress in obesity: Implication for metabolic syndrome, diabetes, hypertension, dyslipidemia, atherosclerosis and cancer. Obes Res Clin Protect.

[19] Kyriakides Z, Kremastinos D, Raptis AE, Johnston N, Raptis SA, Webb DJ (2006). Impaired effect of endothelin-1 on coronary artery stiffness in type 2 diabetes. Int J Cardiol.

[20] Knoir K, Ischibashir M, Yannaji T (1994). Endothelin1 and endothelin 1 in NIDDM patients with and without micro angioplasty. Diabetes Res Clin Pract.

[21] Tarquini B, Perfetto F, Tarquini R, Cornélissen G, Halberg F (1997). Endothelin-1 ‘s chronome indicate diabetic and vascular disease chronorisk. Peptides.

[22] Guillard J, Flavier A, Potier G, Galan P, Hercberg S (2003). Hyperhomocysteinemia: an independent risk factor or a simple marker of vascular disease? 2. Epidemiological data Path Biol.

[23] Drunat S, Moatti N, Demuth K (2002). Homocysteine decreases endothelin-1 expression by interfering with the AP-1 signaling pathway. Free Radic Biol Med.

[24] Rossi G, Giordano A, Breda S, Lisi C, Roura X, Zatelli A (2013). Big-endothelin 1 (big ET-1) and homocysteine in the serum of dogs with chronic kidney disease. Vet J.

